# A Cholesterol Homeostasis-Related Gene Signature Predicts Prognosis of Endometrial Cancer and Correlates With Immune Infiltration

**DOI:** 10.3389/fgene.2021.763537

**Published:** 2021-11-01

**Authors:** Yili Chen, Kaping Lee, Yanchun Liang, Shuhang Qin, Yuan Zhu, Junxiu Liu, Shuzhong Yao

**Affiliations:** ^1^ Department of Obstetrics and Gynecology, The First Affiliated Hospital, Sun Yat-sen University, Guangzhou, China; ^2^ State Key Laboratory of Oncology in South China, Collaborative Innovation Center for Cancer Medicine, Sun Yat-sen University Cancer Center, Guangzhou, China; ^3^ Department of Obstetrics and Gynecology, Nanjing Maternity and Child Health Care Hospital, Obstetrics and Gynecology Hospital Affiliated to Nanjing Medical University, Nanjing, China

**Keywords:** endometrial cancer, cholesterol homeostasis, gene signature, prognosis, immune infiltration

## Abstract

**Background:** Endometrial cancer (EC) is one of the most common gynecological malignancies in women. Cholesterol metabolism has been confirmed to be closely related to tumor proliferation, invasion and metastasis. However, the correlation between cholesterol homeostasis-related genes and prognosis of EC remains unclear.

**Methods:** EC patients from the Cancer Genome Atlas (TCGA) database were randomly divided into training cohort and test cohort. Transcriptome analysis, univariate survival analysis and LASSO Cox regression analysis were adopted to construct a cholesterol homeostasis-related gene signature from the training cohort. Subsequently, Kaplan-Meier (KM) plot, receiver operating characteristic (ROC) curve and principal component analysis (PCA) were utilized to verify the predictive performance of the gene signature in two cohorts. Additionally, enrichment analysis and immune infiltration analysis were performed on differentially expressed genes (DEGs) between two risk groups.

**Results:** Seven cholesterol homeostasis-related genes were selected to establish a gene signature. KM plot, ROC curve and PCA in two cohorts demonstrated that the gene signature was an efficient independent prognostic indicator. The enrichment analysis and immune infiltration analysis indicated that the high-risk group generally had lower immune infiltrating cells and immune function.

**Conclusion:** We constructed and validated a cholesterol homeostasis-related gene signature to predict the prognosis of EC, which correlated to immune infiltration and expected to help the diagnosis and precision treatment of EC.

## Introduction

With about 382,000 new cases and 90,000 deaths worldwide in 2018, endometrial cancer (EC) is one of the most common gynecological malignancies in women, accompanied by the increasing incidence and decreasing age of onset (Bray et al., 2018; Siegel et al., 2019). Moreover, the prognosis of EC patients at different stages are obviously different. Patients with early-stage EC usually have a good prognosis, while those with advanced, metastatic or recurrent EC usually have a poor prognosis (Morice et al., 2016; McGunigal et al., 2017; Urick and Bell, 2019). With the rapid development of molecular biology and sequencing technology, some single-gene biomarkers have been unearthed to predict malignant tumors (Shang et al., 2018; Li et al., 2020a; Du et al., 2020). However, the expression of these single genes is often more susceptible to various factors, so their prediction effect did not seem to meet our expectations. Thence, screening multiple genes regulated by the same significant pathway to establish a high-efficiency gene signature may be a means to improve prediction performance.

Cholesterol is an essential component of mammalian membrane structure and plays an important role in maintaining the life activities of cells and the body (Brown et al., 2018; Giacomini et al., 2021). It has been revealed that cholesterol metabolism disorders are bound up with tumor proliferation, invasion and metastasis. The body maintains its own cholesterol homeostasis mainly through the biosynthesis of endogenous cholesterol and the uptake of exogenous cholesterol. A variety of genes associated to cholesterol synthesis and intake were significantly up-regulated in tumor tissues. The cholesterol synthesis rate-limiting enzyme 3-hydroxy-3-methylglutaryl coenzyme A reductase (HMGCR) was up-regulated in many tumors such as gastric cancer, glioma and prostate cancer. Overexpression of HMGCR promoted the proliferation and migration of these tumor cells, while knockdown of it inhibited tumors proliferation and migration. In addition, targeted inhibition of HMGCR has been initially adopted to treat solid cancer, blood cancer and drug-resistant tumors (Chushi et al., 2016; Qiu et al., 2016; Kong et al., 2018; Lee et al., 2018; Yang et al., 2020). Additionally, the expression of low-density lipoprotein receptor (LDLR) that mediates cholesterol uptake was related to the occurrence and development of breast cancer, pancreatic tumors, glioma, and prostate cancer (Guo et al., 2011; Yue et al., 2014; Guillaumond et al., 2015; Gallagher et al., 2017). However, so far, there are no relevant research on the prognostic value of the gene signature related to cholesterol homeostasis in EC.

**FIGURE 1:**
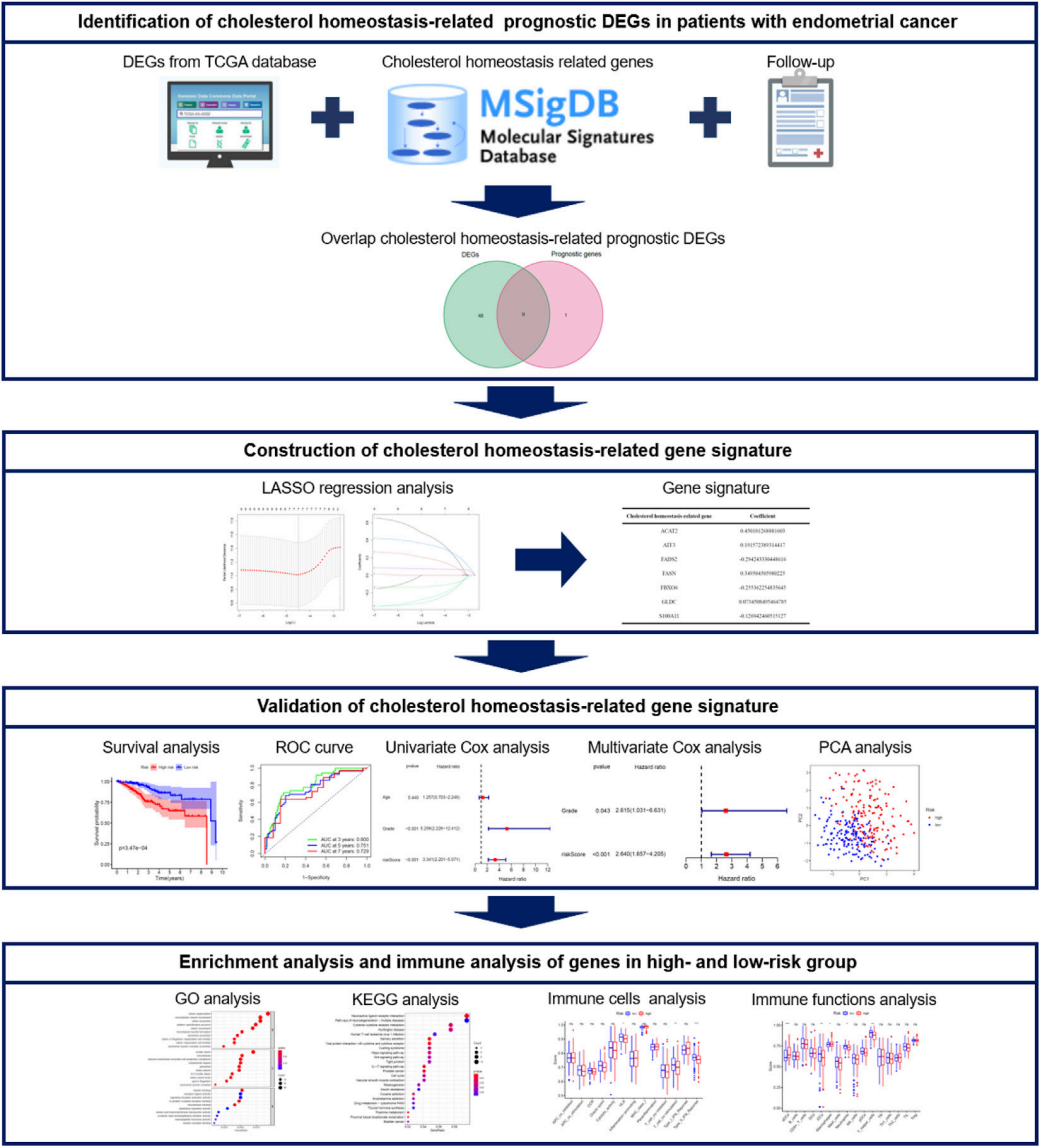
The overall design and flow chart of the study.

In this study, we aim to build an efficient gene signature to predict the prognosis of EC via mining the data in the Cancer Genome Atlas (TCGA). The overview and roadmap of the study are shown in Figure 1. First, we plan to analyze the mRNA expression data and clinical information related to cholesterol homeostasis in EC patients in TCGA, and determine the prognostic differentially expressed genes (DEGs). Then, the least absolute shrinkage and selection operator (LASSO) regression analysis is to be adopted to establish a cholesterol homeostasis related gene signature in the training cohort, whose predictive performance will be further verified through the test cohort. Subsequently, we intend to perform enrichment and immune analysis on DEGs in high- and low-risk groups. In short, we hope to develop an efficient gene signature related to cholesterol homeostasis, which will help the diagnosis and precision treatment of EC.

## Materials and Methods

### Data Collection

The mRNA expression data and clinical information of 552 EC samples and 23 normal samples were downloaded from TCGA database (https://portal.gdc.cancer.gov/). After deleting the samples with missing information and repetitions, using the “sample” function of the R, the remaining 539 EC samples were randomly divided into the TCGA training cohort (*n* = 307) and the TCGA test cohort (*n* = 232) according to the ratio of 6:4. In addition, 74 genes associated to cholesterol homeostasis were sourced from the Molecular Signatures Database (MSigDB) (https://www.gsea-msigdb.org/gsea/msigdb/index.jsp) (Subramanian et al., 2005; Liberzon et al., 2015). The gene list was shown in Supplementary Table S1.

### Identification of Cholesterol Homeostasis-Related Prognostic DEGs

In order to obtain DEGs related to cholesterol homeostasis, we adopted the “limma” R package to analyze the differential expression of mRNA between EC patients and normal patients in the TCGA database with a false discovery rate (FDR) < 0.05. Then, regarded *p* < 0.05 as the critical standard, we performed univariate Cox regression analysis on the overall survival (OS) of 74 cholesterol homeostasis-related genes to obtained the prognostic genes. Subsequently, the DEGs and prognostic genes were intersected to acquire the corresponding prognostic DEGs related to cholesterol homeostasis for further analysis. The “heatmap” R package was utilized to draw a heatmap to more intuitively display the differential expression levels of DEGs between tumor patients and normal patients. In addition, the STRING online tool (http://string-db.org/) and correlation analysis were applied to further explore the relationship between each cholesterol homeostasis-related prognostic DEG.

### Development and Validation of Cholesterol Homeostasis-Related Gene Signature

LASSO regression analysis was employed to construct a prognostic model of cholesterol homeostasis-related genes, and the risk score was calculated by the following formula: risk score = $\overset{n}{\underset{i}{\sum }}Xi\times Yi$ (X: coefficient value of each gene, Y: expression level of each gene). Hereafter, we divided the patients into a high-risk group and a low-risk group based on the median value of the risk score. The Kaplan-Meier (KM) curve and receiver operating characteristic (ROC) curve were used to evaluate the prediction efficiency of cholesterol homeostasis-related gene signature, which was drawn by the “survival” and “timeROC” R packages, respectively. In addition, the principal component analysis (PCA) was introduced to visually demonstrate its predictive performance. Finally, univariate and multivariate Cox regression analysis were applied to further validate the gene signature.

### Enrichment Analysis and Immune Infiltration Analysis

According to median value of the risk score, we divided the EC patients into high- and low-risk groups. Next, regarding |log2FC|≥1 and FDR<0.05 as the specific criteria, we utilized the “limma” R package to screen out the DEGs between the two risk groups. Afterward, we further performed gene ontology (GO) analysis and Kyoto Encyclopedia of Genes and Genomes (KEGG) pathway enrichment analysis for these genes using the “clusterProfiler” R package. Furthermore, we adopted the “gsva” package to perform a single-sample gene set enrichment analysis (ssGSEA) to calculate the scores of immune infiltrating cells and immune function in the TCGA training cohort and TCGA test cohort, and applied the “limma” package to analyze differences in immune scores between high- and low-risk groups.

### Verification of mRNA and Protein Expression of Seven Genes in the Gene Signature

The mRNA expression data of these genes was from the UALCAN database (http://ualcan.path.uab.edu/) (Chandrashekar et al., 2017), while partial representative immunohistochemistry (IHC) pictures of each gene in tumor tissues and normal tissues resourced from the Human Protein Atlas database (https://www.proteinatlas.org/) (Uhlen et al., 2015; Uhlen et al., 2017).

### Mutation Analysis

Furthermore, in order to further explore the mutations of the seven genes in the gene signature, we utilized the cBioportal database (https://www.cbioportal.org/) (Cerami et al., 2012; Gao et al., 2013) to perform mutation analysis on them.

### Statistical Analysis

We adopted Wilcoxon test to analyze the difference in mRNA expression between normal samples and EC samples, while using Mann-Whitney U test to compare the immune scores of EC patients in high- and low-risk groups. In addition, we utilized the KM curve of the two-sided log-rank test to analyze the OS of patients in different risk groups. Univariate and multivariate Cox regression analysis with hazard ratio (HR) and 95% confidence interval (95% CI) were applied to evaluate the predictive power of the gene signature. Unless otherwise specified, *p* < 0.05 is considered statistically significant.

## Results

### Identification of Cholesterol Homeostasis-Related Prognostic DEGs

**FIGURE 2:**
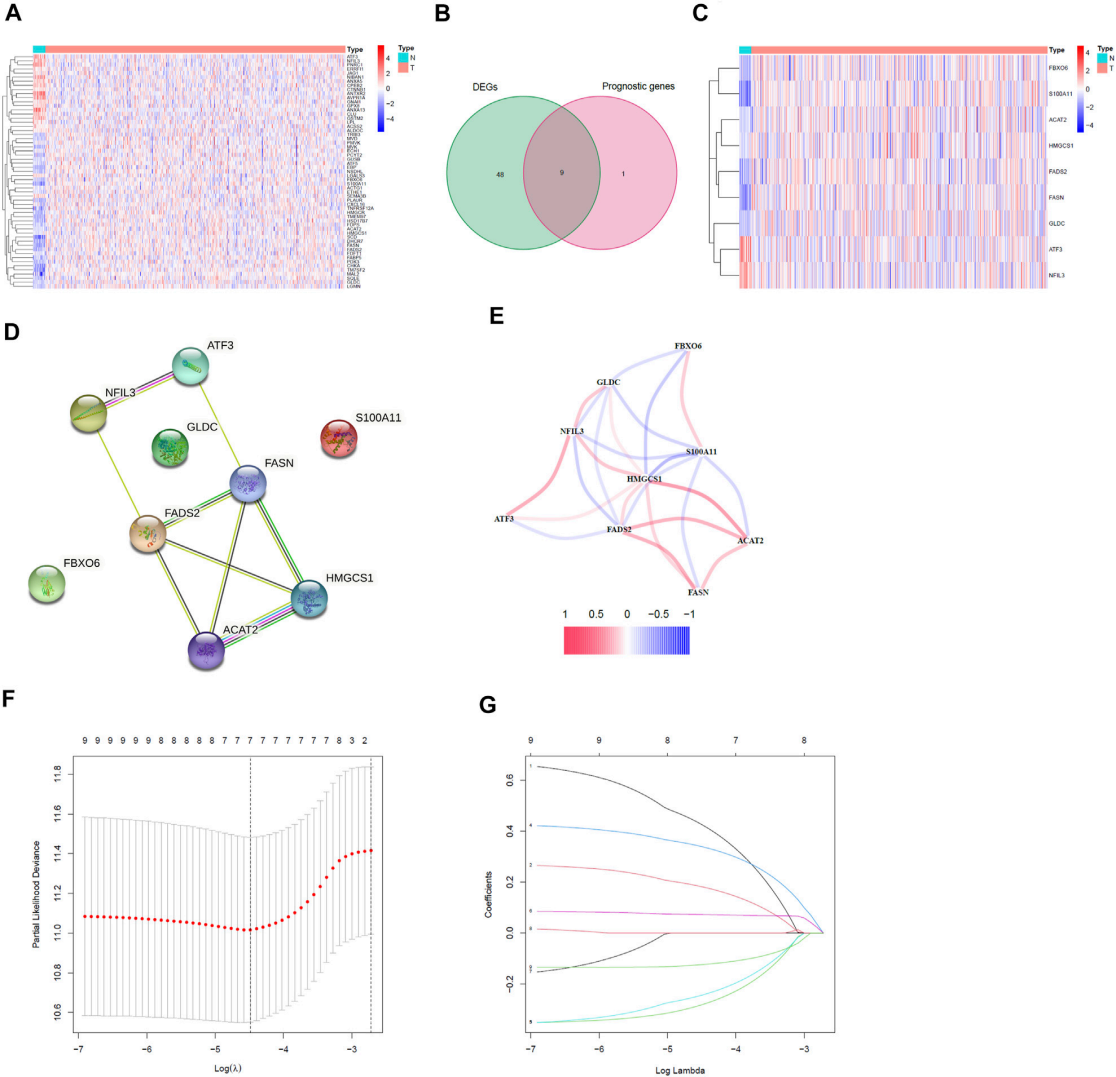
Identification of the prognostic DEGs related to cholesterol homeostasis in EC and the establishment of risk model. **(A)** Heatmap of 57 cholesterol homeostasis-related DEGs. **(B)** Venn diagram between DEGs and prognostic genes. **(C)** Heatmap of nine cholesterol homeostasis related prognostic DEGs. **(D)** The PPI network of prognostic DEGs. **(E)** Correlation analysis of prognostic DEGs. **(F)** The minimum standard and **(G)** the corresponding coefficient of LASSO Cox regression analysis.

We analyzed the expression data of 74 genes related to cholesterol homeostasis in 552 EC samples and 23 normal samples in TCGA database to obtain 57 DEGs (*p* < 0.05, Supplementary Table S2). The heatmap of these genes was shown in Figure 2A. Most of cholesterol homeostasis-related genes were differentially expressed between normal samples and EC samples. Therefore, we could reasonably speculate that cholesterol homeostasis is related to EC. Meanwhile, we screened 74 genes related to cholesterol homeostasis through univariate Cox regression analysis and acquired 10 prognostic genes. Taking the intersection of 57 DEGs and 10 prognostic genes, we got nine cholesterol homeostasis-related prognostic DEGs, namely ACAT2, ATF3, FADS2, FASN, FBXO6, GLDC, HMGCS1, NFIL3 and S100A11 (Figure 2B). Obviously, ATF3 and NFIL3 were down-regulated in tumors, while other seven genes were up-regulated (Figure 2C). Afterward, we conducted a PPI network and correlation analysis on these nine prognostic DEGs, and the results were displayed in Figures 2D,E, respectively.

### Construction of Cholesterol Homeostasis-Related Gene Signature in the TCGA Training Cohort

**FIGURE 3:**
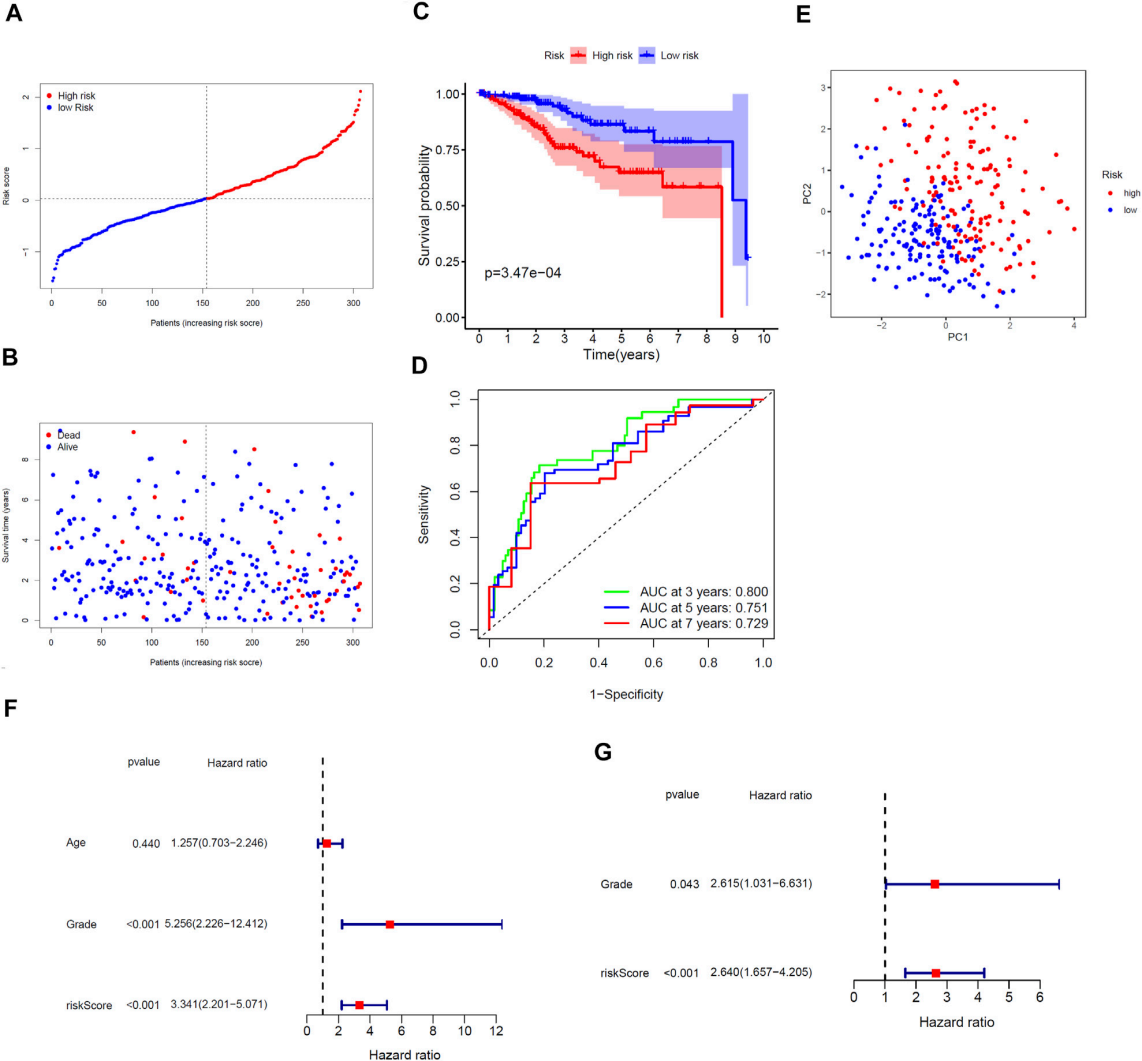
Comprehensive prognostic analysis of the seven-gene signature in the TCGA training cohort. **(A)** Distribution of risk scores for EC patients. **(B)** Distribution of survival time of patients with different risk scores. **(C)** KM plot of OS analysis of EC patients in high- and low-risk groups. **(D)** ROC curve of the gene signature. **(E)** PCA of the gene signature. **(F)** Univariate and **(G)** Multivariate Cox regression analysis.

We performed LASSO regression analysis on the nine prognostic DEGs in the TCGA training cohort and constructed a cholesterol homeostasis-related gene signature composed of seven genes (Figures 2F,G). The risk score formula is as follows: risk score = 0.450×ACAT2 expression value + 0.192×ATF3 expression value-0.294×FADS2 expression value + 0.350×FASN expression value-0.255×FBXO6 expression value + 0.073×GLDC expression value-0.127×S100A11 expression value. The detail coefficient value of each gene in the gene signature was listed in Table 1. Based on the median value of risk score, we divided all tumor samples into a high-risk group (*n* = 153) and a low-risk group (*n* = 154) (Figure 3A). The scatter plot demonstrated that the higher the risk score, the shorter the survival time of patients and the greater the number of deaths (Figure 3B). In addition, the KM curve revealed that the prognosis of the low-risk group was better than that of the high-risk group (*p* < 0.001, Figure 3C). The area under the ROC curve (AUC) for 3, 5, and 7 years were 0.800, 0.751 and 0.729, respectively, indicating the gene signature has a high prediction accuracy (Figure 3D). Furthermore, we explored the predictive power of the gene signature through PCA and found that the two risk groups of patients can be well distributed in the two clusters (Figure 3E). It is worth noting that the univariate and multivariate Cox regression analysis with HR = 3.341 and HR = 2.640 indicated that the risk score is an efficient independent prognostic indicator (*p* < 0.001, Figures 3F,G). Furthermore, we analyzed the relationship between the risk score and clinical characteristics and clarified that the risk score was significantly correlated with age (*p* < 0.0359), grade (*p* < 0.0001), vital status (*p* < 0.0001) and survival time (*p* = 0.0126) (Table 2).

**TABLE 1:** Seven cholesterol homeostasis-associated genes and their coefficient value.

Cholesterol homeostasis-related gene	Coefficient
ACAT2	0.450101268881603
ATF3	0.191572389314417
FADS2	−0.294243330448616
FASN	0.349504505980225
FBXO6	−0.255362254835645
GLDC	0.0734508495464785
S100A11	−0.126942460515127

**TABLE 2:** Correlation between risk score and clinical variables of patients with EC.

Clinical variables	TCGA training cohort			*p*-value	TCGA test cohort			*p*-value
	Total (*n* = 307)	Risk score			Total (*n* = 232)	Risk score		
		High	Low			High	Low	
Age (years)								
≤60	108	45	63		98	50	48	
>60	198	107	91		133	80	53	
Unknown	1	1	0	**0.0359** ^*^	1	1	0	**0.7035**
Grade								
Low (G1 & G2)	126	27	99		91	25	66	
High (G3 & G4)	181	126	55	**<0.0001** ^****^	141	106	35	**<0.0001** ^****^
Vital status								
Alive	259	120	139		193	102	91	
Dead	48	33	15	**<0.0001** ^****^	39	29	10	**0.0960**
Survival time (years)								
≤3	196	105	91		134	82	52	
>3	111	48	63	**0.0126** ^*^	98	49	49	**0.7794**

“*” represents *p* < 0.05.

“****” represents *p* < 0.0001.

Bold values represent *p* < 0.05.

### Validation of the Gene Signature in the TCGA Test Cohort

**FIGURE 4:**
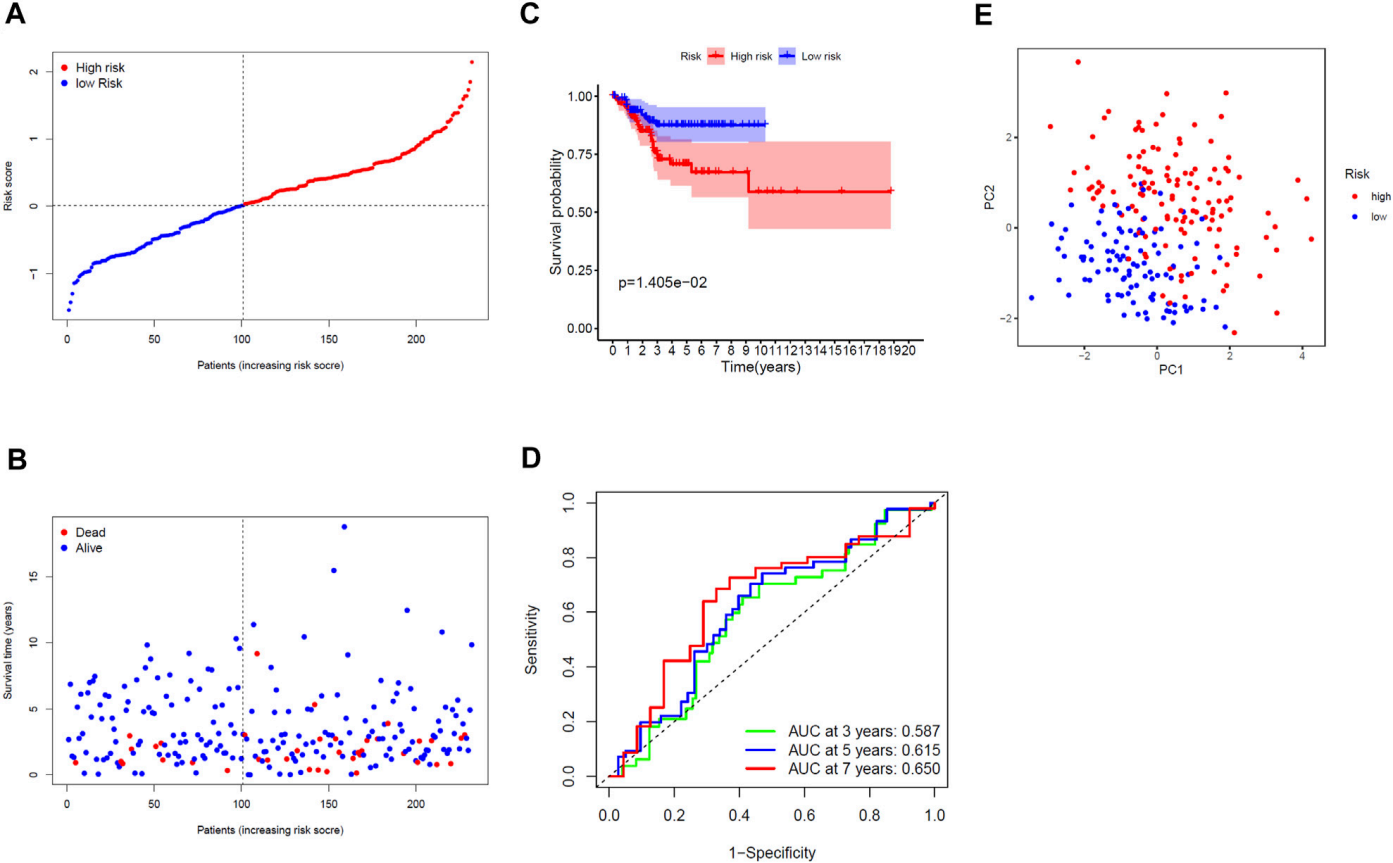
Validated prognostic analysis of the seven-gene signature in the TCGA test cohort. **(A)** Distribution of risk scores for EC patients. **(B)** Distribution of survival time with different risk scores. **(C)** KM curve for EC patients in the high- and low-risk group. **(D)** ROC curve of the gene signature. **(E)** PCA diagram for EC patients.

The 232 patients in the TCGA test cohort were divided into a high-risk group (*n* = 131) and a low-risk group (*n* = 101) (Figure 4A). As the risk score increased, the survival time of patients was shortened and the number of deaths increased (Figure 4B). The KM curve indicated that the prognosis of the high-risk group was significantly worse than that of the low-risk group (*p* = 0.014, Figure 4C). The AUC of ROC curves (0.587 for 3 years, 0.615 for 5 years and 0.650 for 7 years) further verified the prediction accuracy of gene signature (Figure 4D). The PCA for the TCGA test cohort demonstrated that patients in the high- and low-risk groups can also be well distributed in the two clusters, reflecting the predictive stability of the gene signature (Figure 4E). Additionally, exploring the correlation between risk score and clinical variables, we found that the risk score of the gene signature in the TCGA test cohort was significantly correlated with grade (*p* = 0.0001) (Table 2).

### Enrichment Analysis of DEGs in Two Risk Groups

**FIGURE 5:**
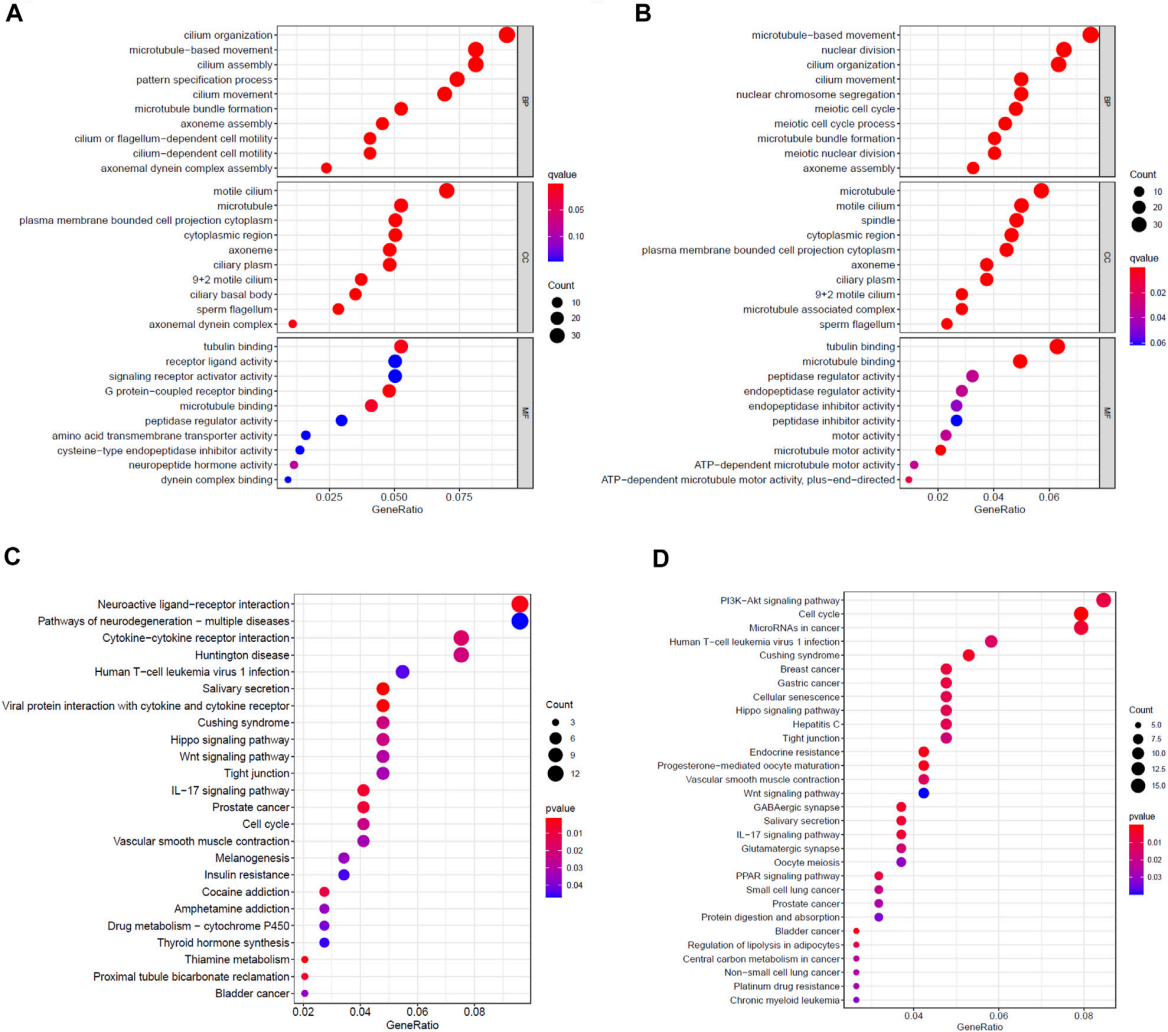
Enrichment analysis of DEGs between high- and low-risk groups. Bubble graphs of **(A)** GO enrichment analysis and **(C)** KEGG pathway enrichment analysis in the TCGA training cohort. Bubble graphs of **(B)** GO enrichment analysis and **(D)** KEGG pathway enrichment analysis in the TCGA test cohort.

First, we analyzed the mRNA expression data of patients in the high- and low-risk groups in the TCGA training cohort, and obtained 624 DEGs (Supplementary Table S3). Then, we performed GO and KEGG enrichment analysis on these DEGs. The results indicated that GO analysis mainly focused on motile cilium, cilium movement, microtubule bundle formation, axoneme assembly and cilium organization (Figure 5A), while KEGG pathway was significantly enriched in salivary secretion, viral protein interaction with cytokine and cytokine receptor, thiamine metabolism, neuroactive ligand-receptor interaction and IL-17 signaling pathway (Figure 5C). Furthermore, we did the same analysis on the TCGA test cohort, and acquired 737 DEGs (Supplementary Table S4). The GO analysis result of the test cohort was very analogous to the training cohort (Figure 5B). However, the KEGG analysis of the test cohort was mainly enriched in cell cycle, endocrine resistance, progesterone-mediated oocyte maturation, bladder cancer and Cushing syndrome, which was quite different from those of the training cohort (Figure 5D).

### Comparison of Immune Cells and Immune Function of EC Patients in High- and Low-Risk Groups

**FIGURE 6:**
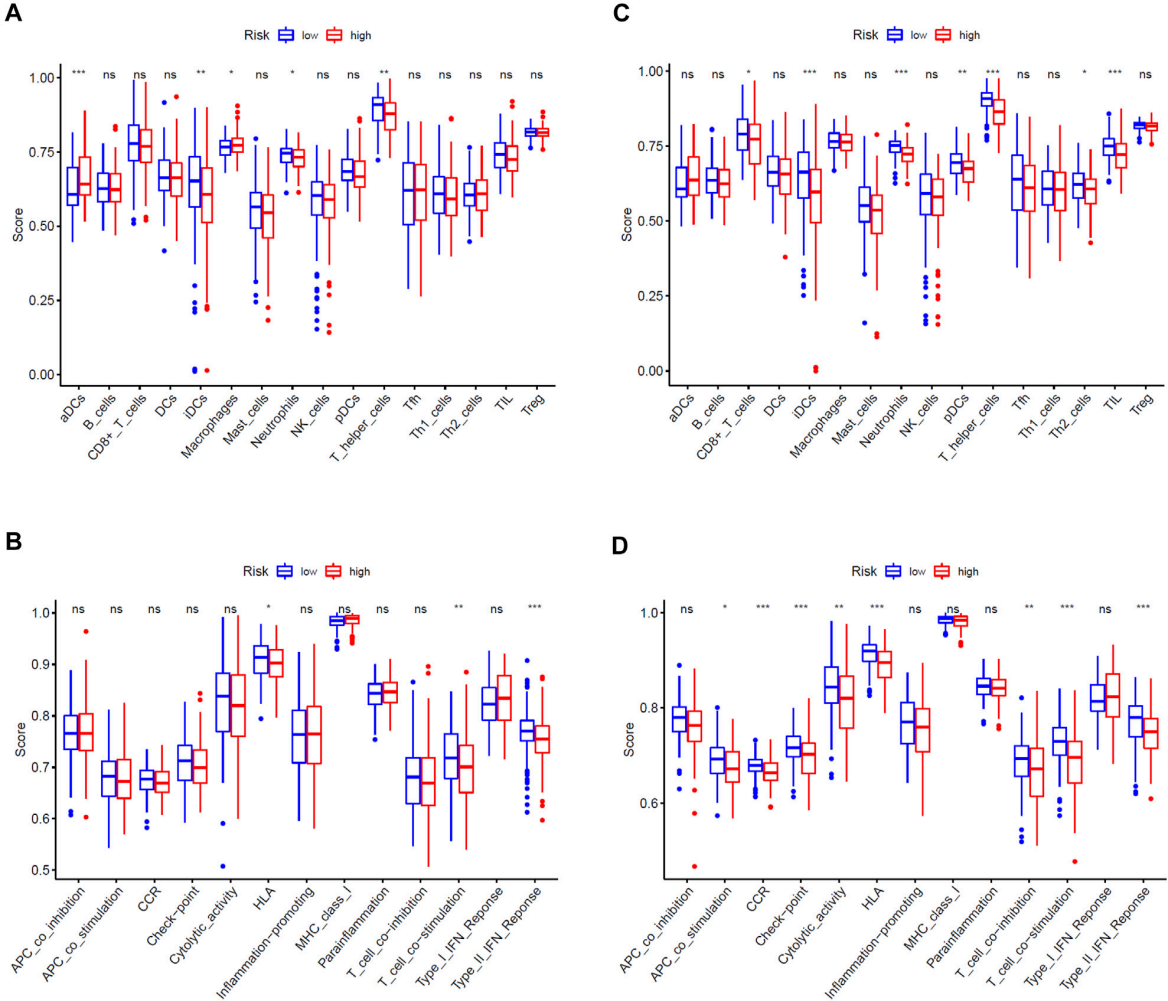
Comparison of immune cells and immune functions of EC patients between high- and low-risk groups. **(A,B)** Comparison of the ssGSEA scores of immune cells and immune functions between high- and low-risk groups in the TCGA training cohort. **(C,D)** Comparison of the ssGSEA scores of immune cells and immune functions between high- and low-risk groups in the TCGA test cohort. The statistical differences were shown as follow: ns, not significant; *, *p* < 0.05; **, *p* < 0.01; ***, *p* < 0.001.

We adopted ssGSEA to score the immune infiltrating cells and immune function of patients in the high- and low-risk groups in the training cohort and test cohort, and analyzed the differences of the scores. Comprehensive analysis of two cohorts, we could disclosure that the immune infiltrating cells and immune function enrichment scores between the high- and low-risk groups are quite different. In general, the immune infiltrating cells and immune function scores of the high-risk group were lower than those of the low-risk group, especially in the test cohort (Figures 6A–D). In detail, the immune infiltrating cells such as immature dendritic cells (iDCs), neutrophils and T helper cells of two cohorts in the high-risk group were significantly lower than those in the low-risk group (Figures 6A,C), while immune function activities such as the type II interferon (IFN) response, T cell co-stimulation and human lymphocyte antigen (HLA) were weaker than the low-risk group (Figures 6B,D).

### Verification of mRNA and Protein Expression of Seven Genes in the Gene Signature

**FIGURE 7:**
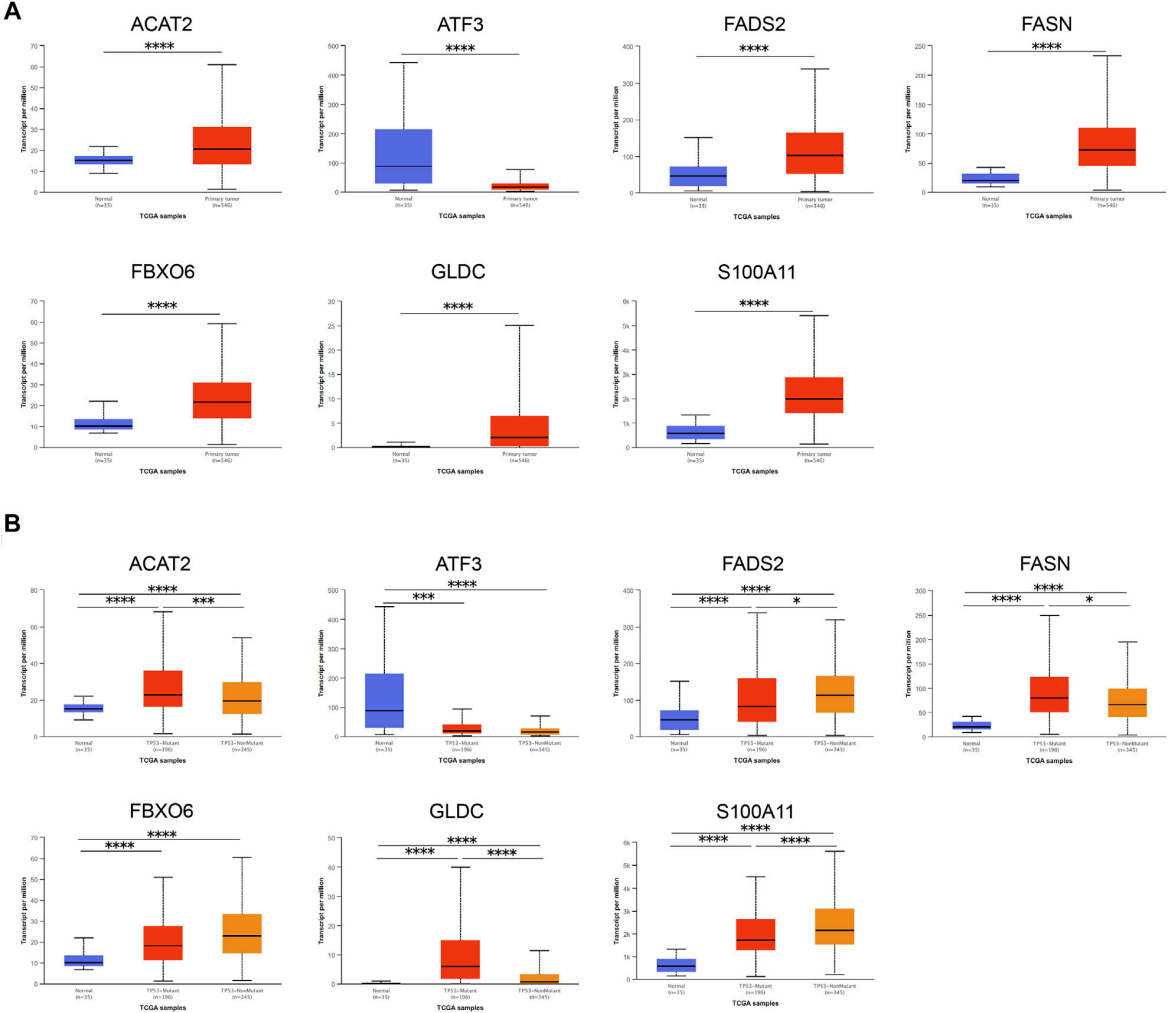
Verification of mRNA expression of seven genes in the gene signature. **(A)** The mRNA expression levels of each gene between EC samples and normal samples. **(B)** The relationship between the mRNA expression and the mutation of TP53 of various genes in EC samples and normal samples. Data was acquired from the UALCAN. *, *p* < 0.05; ***, *p* < 0.001; ****, *p* < 0.0001.

**FIGURE 8:**
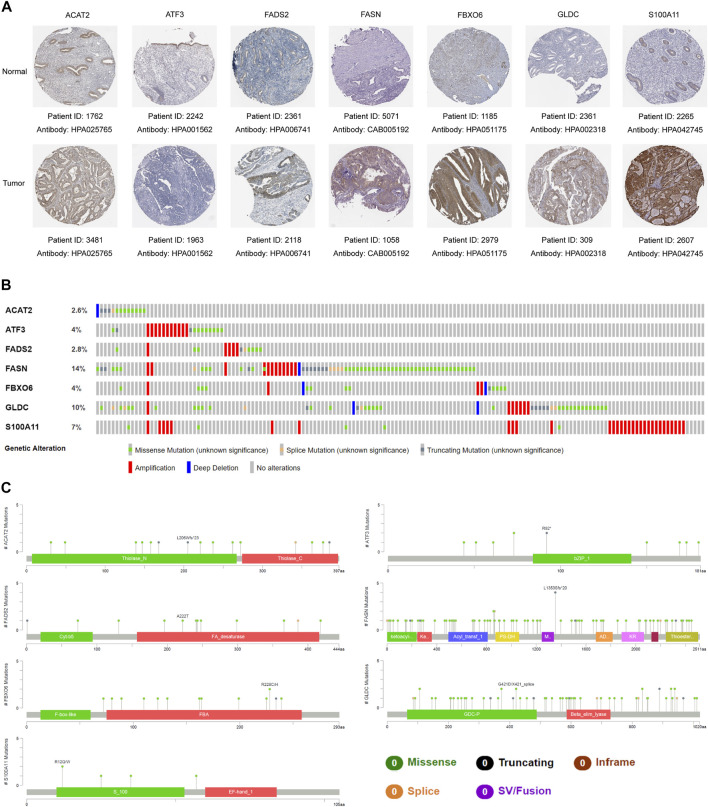
Representative protein expression and mutation analysis of each gene. **(A)** Representative IHC results of each gene in tumor tissues and normal tissues. Data resourced from The Human Protein Atlas. **(B)** Overview of mutations of each gene in the gene signature. **(C)** Detailed mutation sites and types of each gene in the gene signature. The data was derived from the cBioportal database.

First, we applied UALCAN to verify mRNA expression of seven genes in the gene signature, and found the expression between normal samples and tumor samples was significantly different. Compared with normal samples, except for the down-regulation of ATF3, other genes were up-regulated in tumor tissues (Figure 7A). Interestingly, except for ATF3 and FBXO6 (*P*_*ATF3*_ = 0.109, *P*_*FBXO6*_ = 0.051), the expression of other five genes in the gene signature were closely bound up with the mutation of TP53 (Figure 7B). In addition, we adopted representative IHC results in the Human Protein Atlas database to verify that the protein expression levels of these genes are highly consistent with mRNA expression (Figure 8A).

### Mutation Analysis of Seven Genes in the Gene Signature

To further explore whether genes in the gene signature are prone to mutations, we utilized the cBioportal database to analyze the mutations of each gene in EC samples in the TCGA database. It was found that the mutation frequency of these genes was between 2.6% and 14%, mainly missense mutation and amplification. Among them, FASN had the highest mutation frequency at 14%, while ACAT2 had the lowest at only 2.6% (Figure 8B). The detailed mutation types and locations of each gene were displayed in Figure 8C.

## Discussion

With the increasing incidence and decreasing age of onset, EC is still one of the most common intractable gynecological malignancies in women (Bray et al., 2018; Siegel et al., 2019). Patients with early-stage EC generally have a better prognosis, while those with advanced, recurrent or metastatic EC have a worse prognosis. At present, the diagnosis of EC based on clinical symptoms, cytology and transvaginal ultrasound does not seem to be very specific (Kinde et al., 2013; Clarke et al., 2018). With the rapid development of molecular genetics and sequencing technology, some single-gene biomarkers have been unearthed to predict prognosis of malignant tumors (Shang et al., 2018; Li et al., 2020a; Du et al., 2020). However, since they are more susceptible to various factors, the prediction effect of these single gene did not seem to meet our expectations. Therefore, screening for more stable gene signature composed of multiple genes may be a means to improve this dilemma.

Cholesterol homeostasis is essential for maintaining the vital activities of cells and the body. The imbalance of cholesterol homeostasis is closely related to tumor proliferation, invasion and metastasis. It has been demonstrated that cholesterol homeostasis related genes were abnormally expressed in gastric cancer, glioma, prostate cancer, breast cancer and pancreatic tumors (Guo et al., 2011; Yue et al., 2014; Guillaumond et al., 2015; Chushi et al., 2016; Qiu et al., 2016; Gallagher et al., 2017; Kong et al., 2018; Lee et al., 2018; Yang et al., 2020). However, there is no relevant research on the prognostic value of the gene signature related to cholesterol homeostasis in EC so far.

In our study, we have successfully established and verified the gene signature related to cholesterol homeostasis composed of seven genes (ACAT2, ATF3, FADS2, FASN, FBXO6, GLDC and S100A11), which could predict the prognosis of EC.

ACAT2, the full name is Acetyl-CoA Acetyltransferase 2, whose encoded cytoplasmic acetoacetyl-CoA thiolase is an enzyme involved in lipid metabolism. It mainly participates in the biosynthetic pathway of cholesterol (Chang et al., 2009). It has been confirmed that ACAT2 could promote the proliferation, migration and invasion of breast cancer cells after being up-regulated by leptin, which might be a potential biomarker and precision treatment target (Huang et al., 2017).

ATF3, called activating transcription factor 3, is a transcription factor that plays an important role in metabolic regulation, immune response and tumorigenesis (Ku and Cheng, 2020). As the center of the adaptive cellular response network, ATF3 participated in tumorigenesis of colon cancer, breast cancer, prostate cancer, liver cancer and lung cancer (Taketani et al., 2012; Wang and Yang, 2013; Wolford et al., 2013; Li et al., 2017; Li et al., 2019). Thence, as one of the main regulators of metabolic homeostasis, ATF3 may be a valuable target for precise treatment of metabolic imbalance, immune disorders and cancer.

Fatty acid desaturase 2 (FADS2) is the rate-limiting enzyme of fatty acid synthesis. It plays an important role in the reprogramming of fatty acid metabolism and is closely related to the proliferation, invasion and metastasis of tumor cells. It has been found that FADS2 was abnormally expressed in liver cancer, glioblastoma, lung cancer and breast cancer, which may be an important biomarker of tumor metabolic reprogramming (Lane et al., 2003; Wang et al., 2018; Vriens et al., 2019; Li et al., 2020b).

Fatty acid synthase (FASN) is one of the key enzymes in fatty acid synthesis pathway. Past studies have verified that FASN overexpression promoted the occurrence, development and metastasis of tumor cells, which may be a biomarker for many tumors to produce malignant phenotypes and poor prognosis. Besides, recent studies have indicated FASN may play an important role in regulating the expression of pro-apoptotic proteins and DNA repair pathways (Wu et al., 2014; Fhu and Ali, 2020).

The F-box protein6 (FBXO6), also known as FBG2, is a key component of the ubiquitin-protein ligase complex and is widely distributed in most tissues of the human body (Cai et al., 2019). Ji et al. discovered that FBXO6 was significantly highly expressed in ovarian tumor tissues and correlated with the poor prognosis of patients with advanced ovarian tumors (Ji et al., 2021). Cai et al. clarified that high expression of FBXO6 could inhibit the proliferation of non-small cell lung cancer, promote apoptosis and increase the sensitivity of cisplatin (Cai et al., 2019).

Glycine decarboxylase (GLDC) is an oxidoreductase that regulates glycolysis and methylglyoxal metabolism in the glycine cleavage system. It has been reported that GLDC is an oncogene in non-small cell lung cancer and glioma, while other studies have confirmed it is a tumor suppressor in gastric cancer and liver cancer. In short, GLDC is involved in the occurrence and development of several cancers and may be a potential biomarker (Berezowska et al., 2017; Zhuang et al., 2018; Kang et al., 2019; Zhuang et al., 2019).

S100A11 is a member of the S100 protein family which is a multi-gene calcium binding family. Abnormally expressed of S100 protein has been confirmed to be associated to inflammation, cardiomyopathy, neurodegenerative diseases, immune diseases and cancer. Among them, for cancer, it was particularly related to the occurrence and prognosis of bladder cancer, pancreatic cancer, colorectal cancer, esophagus cancer and gastric cancer, and is expected to be an energetic tumor biomarker (Ji et al., 2004; Ohuchida et al., 2006; Salama et al., 2008; Cui et al., 2021; Ma et al., 2021).

In short, these genes are mainly related to cell metabolism, including cholesterol metabolism, fatty acid metabolism, glycolysis and glycine metabolism. However, how they interact with each other remains to be further studied.

Moreover, as mentioned before, in Figure 7B we found that most of cholesterol homeostasis related genes in the gene signature were closely bound up with the mutation of TP53. Jiang et al. revealed that the abnormal amplification of TP53 in HCC was related to cholesterol metabolism disorders through genome-wide analysis (Jiang et al., 2020). Freed-Pastor et al. demonstrated that the highly expressed sterol biosynthesis-related genes in breast tumors were bound up with TP53 mutations and statins could inhibit the proliferation of TP53 mutations cancer cells (Freed-Pastor et al., 2012). Hu et al. clarified that mutant TP53 might play an important role in the progress of high-grade serous ovarian cancer by up-regulating the gene expression of key enzymes in fatty acid and cholesterol biosynthesis (Hu et al., 2013). In summary, we can reasonably speculate that there are inextricable links between cholesterol metabolism, TP53 and tumors. However, the mechanism of their interaction awaits further study.

In addition, the immune infiltration analysis of DEGs between high- and low-risk groups revealed that the immune cell infiltration score and immune function score of the high-risk group were generally lower than those of the low-risk group. It could be speculated that the poor prognosis of patients in the high-risk group may be related to the decline of immune cells and function caused by the imbalance of cholesterol homeostasis. According to these results, we can reasonably speculate that cholesterol homeostasis is bound up with the tumor immune microenvironment (TIME).

Our research inevitably has certain limitations. First, it will be better if the predictive performance of gene signature can be verified through RNA sequencing of collected tissue samples. Secondly, we need to further explore the relationship between these genes and TIME in the follow-up.

## Conclusion

In conclusion, we constructed and verified a cholesterol homeostasis-related gene signature composed of seven genes to predict the prognosis of EC, which correlated with immune infiltration. This gene signature provided a new option for the prognosis prediction of EC, which is expected to help the diagnosis and precision therapy of EC.

## Data Availability

The original contributions presented in the study are included in the article/Supplementary Material, further inquiries can be directed to the corresponding authors.

## References

[B1] Berezowska S. Galván J. A. Langer R. Bubendorf L. Savic S. Gugger M. (2017). Glycine Decarboxylase and HIF-1α Expression Are Negative Prognostic Factors in Primary Resected Early-Stage Non-small Cell Lung Cancer. Virchows Arch. 470 (3), 323–330. 10.1007/s00428-016-2057-z 28062918

[B2] Bray F. Ferlay J. Soerjomataram I. Siegel R. L. Torre L. A. Jemal A. (2018). Global Cancer Statistics 2018: GLOBOCAN Estimates of Incidence and Mortality Worldwide for 36 Cancers in 185 Countries. CA: A Cancer J. Clinicians 68 (6), 394–424. 10.3322/caac.21492 30207593

[B3] Brown M. S. Radhakrishnan A. Goldstein J. L. (2018). Retrospective on Cholesterol Homeostasis: The Central Role of Scap. Annu. Rev. Biochem. 87, 783–807. 10.1146/annurev-biochem-062917-011852 28841344 PMC5828883

[B4] Cai L. Li J. Zhao J. Guo Y. Xie M. Zhang X. (2019). Fbxo6 Confers Drug‐sensitization to Cisplatin via Inhibiting the Activation of Chk1 in Non‐small Cell Lung Cancer. FEBS Lett. 593 (14), 1827–1836. 10.1002/1873-3468.13461 31140586

[B5] Cerami E. Gao J. Dogrusoz U. Gross B. E. Sumer S. O. Aksoy B. A. (2012). The cBio Cancer Genomics Portal: An Open Platform for Exploring Multidimensional Cancer Genomics Data: Figure 1. Cancer Discov. 2 (5), 401–404. 10.1158/2159-8290.CD-12-0095 22588877 PMC3956037

[B6] Chandrashekar D. S. Bashel B. Balasubramanya S. A. H. Creighton C. J. Ponce-Rodriguez I. Chakravarthi B. V. S. K. (2017). UALCAN: A Portal for Facilitating Tumor Subgroup Gene Expression and Survival Analyses. Neoplasia 19 (8), 649–658. 10.1016/j.neo.2017.05.002 28732212 PMC5516091

[B7] Chang T.-Y. Li B.-L. Chang C. C. Y. Urano Y. (2009). Acyl-coenzyme A:cholesterol Acyltransferases. Am. J. Physiology-Endocrinology Metab. 297 (1), E1–E9. 10.1152/ajpendo.90926.2008 PMC2711667 19141679

[B8] Chushi L. Wei W. Kangkang X. Yongzeng F. Ning X. Xiaolei C. (2016). HMGCR Is Up-Regulated in Gastric Cancer and Promotes the Growth and Migration of the Cancer Cells. Gene 587 (1), 42–47. 10.1016/j.gene.2016.04.029 27085483

[B9] Clarke M. A. Long B. J. Del Mar Morillo A. Arbyn M. Bakkum-Gamez J. N. Wentzensen N. (2018). Association of Endometrial Cancer Risk with Postmenopausal Bleeding in Women. JAMA Intern. Med. 178 (9), 1210–1222. 10.1001/jamainternmed.2018.2820 30083701 PMC6142981

[B10] Cui Y. Li L. Li Z. Yin J. Lane J. Ji J. (2021). Dual Effects of Targeting S100A11 on Suppressing Cellular Metastatic Properties and Sensitizing Drug Response in Gastric Cancer. Cancer Cel Int. 21 (1), 243. 10.1186/s12935-021-01949-1 PMC8086328 33931048

[B11] Du Q. Wang W. Liu T. Shang C. Huang J. Liao Y. (2020). High Expression of Integrin α3 Predicts Poor Prognosis and Promotes Tumor Metastasis and Angiogenesis by Activating the C-Src/Extracellular Signal-Regulated Protein Kinase/Focal Adhesion Kinase Signaling Pathway in Cervical Cancer. Front. Oncol. 10, 36. 10.3389/fonc.2020.00036 32117712 PMC7033469

[B12] Fhu C. W. Ali A. (2020). Fatty Acid Synthase: An Emerging Target in Cancer. Molecules 25 (17), 3935. 10.3390/molecules25173935 PMC7504791 32872164

[B13] Freed-Pastor W. A. Mizuno H. Zhao X. Langerød A. Moon S.-H. Rodriguez-Barrueco R. (2012). Mutant P53 Disrupts Mammary Tissue Architecture via the Mevalonate Pathway. Cell 148 (1-2), 244–258. 10.1016/j.cell.2011.12.017 22265415 PMC3511889

[B14] Gallagher E. J. Zelenko Z. Neel B. A. Antoniou I. M. Rajan L. Kase N. (2017). Elevated Tumor LDLR Expression Accelerates LDL Cholesterol-Mediated Breast Cancer Growth in Mouse Models of Hyperlipidemia. Oncogene 36 (46), 6462–6471. 10.1038/onc.2017.247 28759039 PMC5690879

[B15] Gao J. Aksoy B. A. Dogrusoz U. Dresdner G. Gross B. Sumer S. O. (2013). Integrative Analysis of Complex Cancer Genomics and Clinical Profiles Using the cBioPortal. Sci. Signal. 6 (269), pl1. 10.1126/scisignal.2004088 23550210 PMC4160307

[B16] Giacomini I. Gianfanti F. Desbats M. A. Orso G. Berretta M. Prayer-Galetti T. (2021). Cholesterol Metabolic Reprogramming in Cancer and its Pharmacological Modulation as Therapeutic Strategy. Front. Oncol. 11, 682911. 10.3389/fonc.2021.682911 34109128 PMC8181394

[B17] Guillaumond F. Bidaut G. Ouaissi M. Servais S. Gouirand V. Olivares O. (2015). Cholesterol Uptake Disruption, in Association with Chemotherapy, Is a Promising Combined Metabolic Therapy for Pancreatic Adenocarcinoma. Proc. Natl. Acad. Sci. USA. 112 (8), 2473–2478. 10.1073/pnas.1421601112 25675507 PMC4345573

[B18] Guo D. Reinitz F. Youssef M. Hong C. Nathanson D. Akhavan D. (2011). An LXR Agonist Promotes Glioblastoma Cell Death through Inhibition of an EGFR/AKT/SREBP-1/LDLR-dependent Pathway. Cancer Discov. 1 (5), 442–456. 10.1158/2159-8290.CD-11-0102 22059152 PMC3207317

[B19] Hu J. Liu Z. Wang X. (2013). Does TP53 Mutation Promote Ovarian Cancer Metastasis to Omentum by Regulating Lipid Metabolism? Med. Hypotheses 81 (4), 515–520. 10.1016/j.mehy.2013.06.009 23880140

[B20] Huang Y. Jin Q. Su M. Ji F. Wang N. Zhong C. (2017). Leptin Promotes the Migration and Invasion of Breast Cancer Cells by Upregulating ACAT2. Cell Oncol. 40 (6), 537–547. 10.1007/s13402-017-0342-8 PMC13001567 28770546

[B21] Ji J. Zhao L. Wang X. Zhou C. Ding F. Su L. (2004). Differential Expression of S100 Gene Family in Human Esophageal Squamous Cell Carcinoma. J. Cancer Res. Clin. Oncol. 130 (8), 480–486. 10.1007/s00432-004-0555-x 15185146 PMC12161862

[B22] Ji M. Zhao Z. Li Y. Xu P. Shi J. Li Z. (2021). FBXO6-mediated RNASET2 Ubiquitination and Degradation Governs the Development of Ovarian Cancer. Cell Death Dis. 12 (4), 317. 10.1038/s41419-021-03580-4 33767133 PMC7994844

[B23] Jiang J. Zheng Q. Zhu W. Chen X. Lu H. Chen D. (2020). Alterations in Glycolytic/cholesterogenic Gene Expression in Hepatocellular Carcinoma. Aging 12 (11), 10300–10316. 10.18632/aging.103254 32479426 PMC7346031

[B24] Kang P. J. Zheng J. Lee G. Son D. Kim I. Y. Song G. (2019). Glycine Decarboxylase Regulates the Maintenance and Induction of Pluripotency via Metabolic Control. Metab. Eng. 53, 35–47. 10.1016/j.ymben.2019.02.003 30779965

[B25] Kinde I. Bettegowda C. Wang Y. Wu J. Agrawal N. Shih I.-M. (2013). Evaluation of DNA from the Papanicolaou Test to Detect Ovarian and Endometrial Cancers. Sci. Translational Med. 5 (167), 167ra4. 10.1126/scitranslmed.3004952 PMC3757513 23303603

[B26] Kong Y. Cheng L. Mao F. Zhang Z. Zhang Y. Farah E. (2018). Inhibition of Cholesterol Biosynthesis Overcomes Enzalutamide Resistance in Castration-Resistant Prostate Cancer (CRPC). J. Biol. Chem. 293 (37), 14328–14341. 10.1074/jbc.RA118.004442 30089652 PMC6139550

[B27] Ku H.-C. Cheng C.-F. (2020). Master Regulator Activating Transcription Factor 3 (ATF3) in Metabolic Homeostasis and Cancer. Front. Endocrinol. 11, 556. 10.3389/fendo.2020.00556 PMC7457002 32922364

[B28] Lane J. Mansel R. Jiang W. (2003). Expression of Human delta-6-desaturase Is Associated with Aggressiveness of Human Breast Cancer. Int. J. Mol. Med. 12 (2), 253–257. 10.3892/ijmm.12.2.253 12851727

[B29] Lee J. S. Roberts A. Juarez D. Vo T.-T. T. Bhatt S. Herzog L.-o. (2018). Statins Enhance Efficacy of Venetoclax in Blood Cancers. Sci. Transl. Med. 10 (445), eaaq1240. 10.1126/scitranslmed.aaq1240 29899021 PMC6336198

[B30] Li Q. Wang W. Zhang M. Sun W. Shi W. Li F. (2020a). Circular RNA Circ-0016068 Promotes the Growth, Migration, and Invasion of Prostate Cancer Cells by Regulating the miR-330-3p/BMI-1 Axis as a Competing Endogenous RNA. Front. Cel Dev. Biol. 8, 827. 10.3389/fcell.2020.00827 PMC7479067 32984325

[B31] Li X. Zang S. Cheng H. Li J. Huang A. (2019). Overexpression of Activating Transcription Factor 3 Exerts Suppressive Effects in HepG2 Cells. Mol. Med. Rep. 19 (2), 869–876. 10.3892/mmr.2018.9707 30535500 PMC6323204

[B32] Li X. Zhou X. Li Y. Zu L. Pan H. Liu B. (2017). Activating Transcription Factor 3 Promotes Malignance of Lung Cancer Cells *In Vitro* . Thorac. Cancer 8 (3), 181–191. 10.1111/1759-7714.12421 28239957 PMC5415490

[B33] Li Y. L. Tian H. Jiang J. Zhang Y. Qi X. W. (2020b). Multifaceted Regulation and Functions of Fatty Acid Desaturase 2 in Human Cancers. Am. J. Cancer Res. 10 (12), 4098–4111. 33414988 PMC7783767

[B34] Liberzon A. Birger C. Thorvaldsdóttir H. Ghandi M. Mesirov J. P. Tamayo P. (2015). The Molecular Signatures Database Hallmark Gene Set Collection. Cel Syst. 1 (6), 417–425. 10.1016/j.cels.2015.12.004 PMC4707969 26771021

[B35] Ma G. Dai W. Zhang J. Li Q. Gu B. Song Y. (2021). ELK1-mediated U-pregulation of lncRNA LBX2-AS1 F-acilitates C-ell P-roliferation and I-nvasion via R-egulating miR-491-5p/S100A11 axis in C-olorectal C-ancer. Int. J. Mol. Med. 48 (1). 10.3892/ijmm.2021.4971 PMC8175069 34080639

[B36] McGunigal M. Liu J. Kalir T. Chadha M. Gupta V. (2017). Survival Differences Among Uterine Papillary Serous, Clear Cell and Grade 3 Endometrioid Adenocarcinoma Endometrial Cancers: A National Cancer Database Analysis. Int. J. Gynecol. Cancer 27 (1), 85–92. 10.1097/IGC.0000000000000844 27759595

[B37] Morice P. Leary A. Creutzberg C. Abu-Rustum N. Darai E. (2016). Endometrial Cancer. The Lancet 387 (10023), 1094–1108. 10.1016/s0140-6736(15)00130-0 26354523

[B38] Ohuchida K. Mizumoto K. Ohhashi S. Yamaguchi H. Konomi H. Nagai E. (2006). S100A11, a Putative Tumor Suppressor Gene, Is Overexpressed in Pancreatic Carcinogenesis. Clin. Cancer Res. 12 (18), 5417–5422. 10.1158/1078-0432.CCR-06-0222 17000675

[B39] Qiu Z. Yuan W. Chen T. Zhou C. Liu C. Huang Y. (2016). HMGCR Positively Regulated the Growth and Migration of Glioblastoma Cells. Gene 576 (1 Pt 1), 22–27. 10.1016/j.gene.2015.09.067 26432005

[B40] Salama I. Malone P. S. Mihaimeed F. Jones J. L. (2008). A Review of the S100 Proteins in Cancer. Eur. J. Surg. Oncol. (Ejso) 34 (4), 357–364. 10.1016/j.ejso.2007.04.009 17566693

[B41] Shang C. Wang W. Liao Y. Chen Y. Liu T. Du Q. (2018). LNMICC Promotes Nodal Metastasis of Cervical Cancer by Reprogramming Fatty Acid Metabolism. Cancer Res. 78 (4), 877–890. 10.1158/0008-5472.CAN-17-2356 29229603

[B42] Siegel R. L. Miller K. D. Jemal A. (2019). Cancer Statistics, 2019. CA A. Cancer J. Clin. 69 (1), 7–34. 10.3322/caac.21551 30620402

[B43] Subramanian A. Tamayo P. Mootha V. K. Mukherjee S. Ebert B. L. Gillette M. A. (2005). Gene Set Enrichment Analysis: a Knowledge-Based Approach for Interpreting Genome-wide Expression Profiles. Proc. Natl. Acad. Sci. 102 (43), 15545–15550. 10.1073/pnas.0506580102 16199517 PMC1239896

[B44] Taketani K. Kawauchi J. Tanaka-Okamoto M. Ishizaki H. Tanaka Y. Sakai T. (2012). Key Role of ATF3 in P53-dependent DR5 Induction upon DNA Damage of Human colon Cancer Cells. Oncogene 31 (17), 2210–2221. 10.1038/onc.2011.397 21927023

[B45] Uhlen M. Fagerberg L. Hallstrom B. M. Lindskog C. Oksvold P. Mardinoglu A. (2015). Tissue-based Map of the Human Proteome. Science 347 (6220), 1260419. 10.1126/science.1260419 25613900

[B46] Uhlen M. Zhang C. Lee S. Sjöstedt E. Fagerberg L. Bidkhori G. (2017). A Pathology Atlas of the Human Cancer Transcriptome. Science 357 (6352), eaan2507. 10.1126/science.aan2507 28818916

[B47] Urick M. E. Bell D. W. (2019). Clinical Actionability of Molecular Targets in Endometrial Cancer. Nat. Rev. Cancer 19 (9), 510–521. 10.1038/s41568-019-0177-x 31388127 PMC7446243

[B48] Vriens K. Christen S. Parik S. Broekaert D. Yoshinaga K. Talebi A. (2019). Evidence for an Alternative Fatty Acid Desaturation Pathway Increasing Cancer Plasticity. Nature 566 (7744), 403–406. 10.1038/s41586-019-0904-1 30728499 PMC6390935

[B49] Wang C.-M. Yang W.-H. (2013). Loss of SUMOylation on ATF3 Inhibits Proliferation of Prostate Cancer Cells by Modulating CCND1/2 Activity. Ijms 14 (4), 8367–8380. 10.3390/ijms14048367 23591848 PMC3645748

[B50] Wang J. Liang H. Sun M. Zhang L. Xu H. Liu W. (2018). Delta-6-desaturase Inhibitor Enhances Radiation Therapy in Glioblastoma *In Vitro* and *In Vivo* . Cmar 10, 6779–6790. 10.2147/CMAR.S185601 PMC6289123 30584371

[B51] Wolford C. C. McConoughey S. J. Jalgaonkar S. P. Leon M. Merchant A. S. Dominick J. L. (2013). Transcription Factor ATF3 Links Host Adaptive Response to Breast Cancer Metastasis. J. Clin. Invest. 123 (7), 2893–2906. 10.1172/JCI64410 23921126 PMC3696548

[B52] Wu X. Qin L. Fako V. Zhang J.-T. (2014). Molecular Mechanisms of Fatty Acid Synthase (FASN)-mediated Resistance to Anti-cancer Treatments. Adv. Biol. Regul. 54, 214–221. 10.1016/j.jbior.2013.09.004 24080588

[B53] Yang J. Wang L. Jia R. (2020). Role of De Novo Cholesterol Synthesis Enzymes in Cancer. J. Cancer 11 (7), 1761–1767. 10.7150/jca.38598 32194787 PMC7052851

[B54] Yue S. Li J. Lee S.-Y. Lee H. J. Shao T. Song B. (2014). Cholesteryl Ester Accumulation Induced by PTEN Loss and PI3K/AKT Activation Underlies Human Prostate Cancer Aggressiveness. Cel Metab. 19 (3), 393–406. 10.1016/j.cmet.2014.01.019 PMC3969850 24606897

[B55] Zhuang H. Li Q. Zhang X. Ma X. Wang Z. Liu Y. (2018). Downregulation of glycine Decarboxylase Enhanced Cofilin-Mediated Migration in Hepatocellular Carcinoma Cells. Free Radic. Biol. Med. 120, 1–12. 10.1016/j.freeradbiomed.2018.03.003 29524606

[B56] Zhuang H. Wu F. Wei W. Dang Y. Yang B. Ma X. (2019). Glycine Decarboxylase Induces Autophagy and Is Downregulated by miRNA-30d-5p in Hepatocellular Carcinoma. Cel Death Dis. 10 (3), 192. 10.1038/s41419-019-1446-z PMC6389915 30804330

